# Osteogenic Differentiation Properties of BLGFs/ODEX Hydrogel Doped With Collagen Derived From *Chambo* tilapia Fish Skin

**DOI:** 10.1155/ijbm/8564127

**Published:** 2026-07-31

**Authors:** Bhahat Lawlley Zimba, Xiaohan Yang, Mwemezi Rwiza, Elingarami Sauli, Shenqi Wang

**Affiliations:** ^1^ Advanced Biomaterials and Tissue Engineering Center, College of Life Science and Technology, Huazhong University of Science and Technology (HUST), Wuhan 430074, China, hust.edu.cn; ^2^ School of Materials, Energy, Water and Environmental Sciences (MEWES), Nelson Mandela African Institution of Science and Technology, P.O. Box 444, Arusha, Tanzania, nm-aist.ac.tz; ^3^ Bacteriophage Research Unit, Biomedical Engineering Section, Malawi University of Science and Technology (MUST), P.O. Box 5196, Limbe, Malawi, must.ac.mw; ^4^ School of Life Sciences and Bio-Engineering (LiSBE), The Nelson Mandela African Institution of Science and Technology (NM-AIST), Arusha, Tanzania, nm-aist.ac.tz

**Keywords:** biomaterials, bone tissue engineering, collagen, osteogenic differentiation

## Abstract

**Background:**

Bone defects caused by trauma, congenital disorders and degenerative diseases such as osteoporosis remain a major clinical challenge, especially in developing regions. Although bone grafting is the current gold standard for treating bone defects, it faces limitations, including donor site morbidity, limited availability and immune rejection. This study developed and evaluated a novel hybrid biomaterial composed of bovine β‐lactoglobulin fibrils (BLGFs), oxidized dextran (ODEX) and collagen (COL) extracted from *Chambo* tilapia fish skin for bone tissue engineering applications.

**Methods:**

BLGFs were synthesized from whey protein, while ODEX was produced by oxidizing dextran. Three biomaterials–COL, BLGFs/ODEX and BLGFs/ODEX/COL–were synthesized and characterized using Fourier‐transformed infrared (FTIR), scanning electron microscope (SEM), and X‐ray diffraction (XRD). Cytocompatibility and haemocompatibility were assessed through MTT and haemolysis assays using rat red blood cells. Osteogenic potential was evaluated by Alizarin Red staining after culturing rat bone marrow stem cells in osteoinduction media.

**Results:**

The BLGFs/ODEX/COL hydrogel exhibited better porosity, biocompatibility and mechanical stability. SEM and MTT results confirmed robust cell adhesion, spreading, and viability, while haemolysis rates remained below 5%. Enhanced calcium deposition in rBMSCs indicated strong osteogenic differentiation. These results demonstrate that the BLGFs/ODEX/COL hydrogel is a promising, affordable, and biocompatible scaffold for bone tissue regeneration.

## 1. Introduction

Bone tissue plays a vital role in structural support, protection of internal organs, mineral homeostasis and haematopoiesis. However, bone defects that result from trauma, tumour resection, congenital anomalies and degenerative diseases such as osteoporosis present significant clinical challenges. Globally, millions of individuals suffer from bone‐related injuries and defects annually, with the World Health Organization (WHO) projecting that musculoskeletal conditions will be the leading contributor to physical disability worldwide by 2030 [1]. Among these conditions, fractures and bone loss due to osteoporosis affect over 200 million people globally, particularly elderly populations and postmenopausal women [2]. The increasing life expectancy and ageing population have amplified the prevalence and socioeconomic burden of these conditions, driving the need for advanced strategies for bone repair and regeneration.

In sub‐Saharan Africa, the burden of bone injuries and degenerative bone diseases is further exacerbated by factors such as high rates of road traffic accidents, occupational injuries, limited access to specialized orthopaedic care and inadequate infrastructure for trauma management. Additionally, nutritional deficiencies, delayed diagnosis and high infection rates compromise the natural healing processes, making bone regeneration more complex [3]. The rising incidence of osteoporosis and bone injuries in both urban and rural populations has placed significant strain on healthcare systems in developing countries in this region, which often lack enough resources and equipment to provide efficient surgical interventions or rehabilitation at a large scale.

Autologous bone grafting is currently the clinical gold standard for treating large bone defects [4]. The main advantages of autologous bone grafting are that it offers osteoconductivity, osteoinductivity and osteogenic cells. However, it is limited by donor site morbidity, insufficient supply for large defects and the need for additional surgery [5, 6]. As such, allografts, which are derived from cadaveric donors, are commonly used when autologous bone grafting is not feasible because allografts can circumvent some of the limitations of autologous bone grafting [7]. Allografts also have shortfalls, as they do pose a risk of immune rejection, disease transmission and inconsistent integration with host tissue. To address these limitations of autologous and allograft bone grafting, other techniques, like the use of bone substitutes (e.g., calcium phosphates, hydroxyapatite [HA] and tricalcium phosphate), metallic implants and synthetic polymers, have been employed to treat bone injuries and defects in hospitals [8]. While these treatment options provide mechanical support and scaffold structures, most of them lack biological cues that are necessary for promoting cellular activities for bone tissue regeneration. This gap has driven research on suitable biomaterials for bone tissue repair that can combine structural strength with biological functionality.

A wide range of biomaterials have been investigated for bone tissue engineering applications. These biomaterials can be synthesized from natural polymers (e.g., collagen, gelatin, alginate, chitosan and silk fibroin), synthetic polymers (e.g., poly(lactic‐co‐glycolic acid) [PLGA] and poly(ethylene glycol) [PEG]) and bioactive ceramics (e.g., HA, bioactive glass and biphasic calcium phosphate). Natural polymers, especially collagen and gelatin, closely mimic the native extracellular matrix (ECM) of bone tissue, and they provide excellent biocompatibility and cell‐adhesion sites [9]. However, they often lack mechanical robustness to effectively substitute autologous bone grafts. On the other hand, synthetic polymers offer tunable properties and structural integrity but may lack functional features for bone repair or might even elicit inflammatory responses [10].

In our previous work, we successfully fabricated and characterized the physicochemical properties of BLGFs/ODEX/COL hydrogels, which confirmed that the materials are suitable for bone tissue engineering applications [11]. Building on these findings, the present study further investigates the osteogenic differentiation ability of the BLGFs/ODEX/COL hydrogels. In this study, bovine β‐lactoglobulin fibrils (BLGFs), oxidized dextran (ODEX) and collagen were combined to synthesize a biomaterial that can have applications in bone tissue engineering. BLGFs are a biocompatible, protein‐based nanomaterial derived from whey protein in bovine milk. BLGFs exhibit desirable physicochemical properties, including excellent mechanical strength, biodegradability, the ability to support cell adhesion and the capacity to form hydrogels. ODEX was synthesized through the controlled oxidation of dextran, introducing aldehyde groups that facilitate hydrogel formation via Schiff base reactions. Collagen was extracted from *Chambo* (*Oreochromis lidole*) tilapia fish skin. Collagen is a natural polymer that also forms a greater proportion of body tissues, including bone tissue. The combination of these biomaterials offers favourable attributes, including biodegradability, biocompatibility, tunable mechanical strength and controlled release kinetics, making them an excellent candidate for bone tissue engineering applications.

## 2. Materials and Methods

### 2.1. Materials

Luria–Bertani (LB) broth was purchased from Hopebio (China). Whey protein isolate (HilmarTM 9400) was purchased from Hilmar Industries (USA). Dextran (70 kDa) was procured from Aladdin Co., Ltd (China). Foetal bovine serum (FBS) was purchased from Thermo Fisher Scientific (USA). All cell experiments were done in triplicate (*n* = 3) to ensure the findings are consistent, accurate, and statistically valid. Therefore, data are presented as the mean values from the triplicates. GraphPad Prism software (Version 10.4.2) and Microsoft Excel were used to analyse the raw data and produce graphs.

### 2.2. Preparation of BLGFs

β‐Lactoglobulin monomers (BLGMs) are used in the preparation of BLGFs. To prepare BLGMs, whey protein isolate powder (60 g) and sodium citrate tribasic dihydrate (45 g) were dissolved in 1000 mL of Milli‐Q water under continuous stirring until fully dissolved. The pH of the solution was adjusted to 3.9 by adding citric acid monohydrate gradually to the mixture before incubating it for 1 h at 35°C. After incubation, the mixture was centrifuged at 10,000 rpm for 10 min at 4°C. The supernatant was collected and mixed with 140 g sodium chloride (NaCl), and the mixture was stirred for 1 h. Thereafter, the mixture was centrifuged again under the same conditions (10,000 rpm for 10 min at 4°C), and the resulting supernatant was subjected to dialysis against Milli‐Q water at 4°C for 48 h. The dialysed solution was then filtered using a 0.22 μm Millipore filter and further dialysed for an additional 48 h at 4°C. Then the solution was filtered again and lyophilized for 72 h to obtain BLGMs, which were later stored at −80°C for subsequent experiments.

To prepare BLGFs, BLGMs underwent modification under high temperatures and acidic conditions. Briefly, 0.4 g of BLG monomers were dissolved in 9.6 mL of Milli‐Q water, and the pH was adjusted to 2.0 using hydrochloric acid (HCl). The mixture was then filtered and subsequently heated at 90°C for 5 h under continuous stirring. After incubating for 5 h, BLGMs were successfully converted into BLGFs (4 wt%). The reaction was terminated by immediately cooling the mixture on ice. The synthesized BLGFs were stored at 4°C for use in subsequent experiments.

### 2.3. Preparation of ODEX

ODEX was synthesized by oxidizing dextran. The process involved dissolving 5 g of dextran into 125 mL of Milli‐Q water under continuous stirring until the dextran was fully solubilized. To initiate the oxidation reaction, 1 g of sodium periodate (NaIO_4_) was added to the solution and the reaction mixture was incubated for 3.5 h at 35°C. To terminate the oxidation reaction, 6 mL of ethylene glycol was added to the solution. The resulting solution was subjected to dialysis using Milli‐Q water for 72 h at 4°C to remove residual reagents. After dialysis, the solution was lyophilized to obtain ODEX powder. ODEX powder was then stored at −80°C for use in subsequent experiments.

### 2.4. Preparation of Collagen

The acid‐soluble collagen (COL) was extracted from *Chambo* tilapia fish (*Oreochromis lidole*) skin. The fish skin was initially shade‐dried before being cut into small pieces. The fish skin pieces were then immersed in 0.5 M acetic acid containing 5 mM ethylenediaminetetraacetic acid (EDTA). The pH was adjusted to 2.5 before incubating the mixture at 4°C for 96 h. After soaking, the fish skin residues were removed, and the resulting solution was filtered to obtain a collagen‐rich filtrate. Collagen was precipitated from the filtrate by salting out with 4 M NaCl. The precipitate then underwent dialysis against Milli‐Q water to remove the residual salts. This was followed by lyophilization to obtain collagen, which was stored at −80°C for use in subsequent experiments.

### 2.5. Preparation of Hydrogel Biomaterials

A 4 wt% BLGF solution was prepared by dissolving BLGF in Milli‐Q water. The pH of the BLGF solution was adjusted to 7.4 using 10 M sodium hydroxide (NaOH) solution. A different solution was prepared by dissolving ODEX powder in Milli‐Q water at a concentration of 12 wt% ODEX solution. A volume of 4.5 mL from the BLGF solution was then mixed with 1.5 mL of the ODEX solution in a tube. After 2–5 min, the mixture was converted into BLGF/ODEX hydrogel. To synthesize the BLGF/ODEX/Col hydrogel biomaterial, 0.01 g of Col was added to BLGF/ODEX before the solution was converted into a hydrogel. The prepared hydrogel biomaterials were then lyophilized and stored at −80°C for use in subsequent experiments.

### 2.6. Characterization of the Materials

The prepared materials were characterized using different characterization techniques. The morphological and structural characteristics of the materials were analysed by Scanning Electron Microscopy (FEI Company, Hillsboro, OR) at 10 kV and 3.5 μA. The morphology of rat bone marrow stem cells was observed using a scanning electron microscope (SEM; Nova NanoSEM 450). Visualization of the stained samples was done using a Nikon ECLIPSE Ti‐S Microscope (Nikon Corporation, Japan). The haemolysis test results were analysed using the Eon Biotek microplate spectrophotometer. To analyse the functional groups in the samples, an attenuated total reflection Fourier‐transformed infrared (ATR‐FTIR) spectrum was collected on an FTIR spectrometer (Nicolet iS50R) within the range of 500–4000 cm^−1^. To determine the crystallinity of the materials, an X‐ray diffraction (XRD) spectrum was collected using a Rigaku Smartlab diffractometer in the 2θ range of 5°–80°.

### 2.7. Cell Viability Assay

Rat bone marrow‐derived mesenchymal stem cells (rBMSCs) were seeded in triplicate onto the synthesized hydrogel materials in 24‐well culture plates at a density of 2 × 10^3^ cells/well. Dulbecco’s Modified Eagle’s Medium (DMEM) supplemented with 15% FBS and 1% penicillin/streptomycin was used to culture the rBMSCs. The media were changed every 48 h. The cell viability of the rBMSCs was assessed at different time intervals using the MTT assay (3‐(4,5‐dimethyl‐2‐thiazolyl)‐2,5‐diphenyl‐2H‐tetrazolium bromide). On the designated day of the MTT assay, the growth media were removed from the wells, and 100 μL of MTT solution (0.5 mg/mL) was added before incubating the plates at 37°C for 4 h. Following incubation, the MTT solution was carefully aspirated, and 500 μL of dimethyl sulfoxide (DMSO) was added to each well to dissolve the formazan crystals. Subsequently, 150 μL of the DMSO solution was pipetted from each well into a 96‐well plate, and the optical density (OD) was measured at OD_545_ nm using a microplate reader to quantify cell viability using the absorbance values.

### 2.8. Cell Morphology Analysis

For morphological assessment, rBMSCs were seeded in triplicate onto the synthesized hydrogel materials in 24‐well plates at a density of 2 × 10^3^ cells/well and maintained in growth medium for 7 days. The media was changed every 48 h. After 7 days, the cells were fixed with 4% (v/v) paraformaldehyde for 30 min at room temperature. This was then followed by gradient dehydration using different concentrations of ethanol solutions. Then the hydrogels were freeze‐dried to preserve cellular architecture before scanning electron microscopy (Nova NanoSEM 450) was used to evaluate cell adhesion, spreading, and overall morphology on the surfaces of the synthesized hydrogel materials.

### 2.9. Haemocompatibility of Synthesized Hydrogels

The haemocompatibility of the materials was tested using rat red blood cells (RBCs). The materials (BLGF/ODEX, BLGF/ODEX/COL, and COL) were thoroughly rinsed in a tube using normal saline prior to the assay. Deionized water was used as a positive control, while an untreated RBC suspension was used as a negative control. Subsequently, 1 mL of a 3% (v/v) rat RBC suspension was added to the tubes in triplicate. The tubes were then incubated at 37°C with 5% CO_2_ for 1 h. After incubation, the suspension was collected into 1.5 mL microcentrifuge tubes and centrifuged for 10 min at 1200 rpm. After centrifugation, the tubes were visually inspected for evidence of haemolysis. Thereafter, 100 μL of the supernatant from each tube was transferred into a 96‐well plate. A microplate reader was used to measure the OD of the sample at OD_545_ nm. The following formula was used to calculate the haemolysis rate:

(1) Hr%=At− AnAp− An×100%,

where *H*_*r*_ *is* the haemolysis rate while *A*_*t*_,  *A*_*n*_, and *A*_*p*_ are the absorbance values of the test samples, the negative control, and the positive control, respectively.

### 2.10. In Vitro Analysis of Osteogenic Differentiation Ability of the Hydrogel

To determine the in vitro osteogenic differentiation capability of the synthesized materials, rBMSCs were seeded in triplicates on the pre‐sterilized (using UV light for 48 h) BLGFs‐ODEX, BLGFs‐ODEX‐COL, and COL biomaterials at a density of 2 × 10^3^ cells/well using osteogenic induction medium DMEM supplemented with 15% FBS, 1% double antibiotic (penicillin/streptomycin), 10 nM dexamethasone, 10 mM β‐glycerophosphate, and 0.2 mM ascorbic acid. The rBMSCs cultured using growth media in the absence of biomaterials were used as the control group. The culture plates were incubated at 37°C with 5% CO_2,_ and the osteogenic media were changed every 2 days. After 14 days, the rBMSCs were fixed with 4% (vol/vol) paraformaldehyde for 30 min at room temperature. The fixed cells were then stained with Alizarin Red Solution (1%) for 30 min at 37°C. Thereafter, the stained samples were viewed under a microscope to observe the deposition of calcified tissue.

## 3. Results and Discussion

### 3.1. Structural and Functional Properties of the Hydrogel

The BLGMs were used to synthesize fibrous BLGFs by reassembling the β‐sheet structures of spherical BLGMs under acidic and elevated temperature conditions. The resulting elongated fibrillar BLGF structures serve as the primary scaffold within the BLGFs/ODEX hydrogel and play a critical role in promoting cell adhesion [12–14]. Concurrently, Dextran underwent oxidation in the presence of sodium periodate (NaIO_4_) to yield ODEX with multiple aldehyde groups. The BLGF/ODEX hydrogel was successfully synthesized by adding ODEX to β‐lactoglobulin fibres (at a ratio of 1:3) before allowing the mixture to stand for a few minutes to allow gelation to take place. The synthesis strategy and the physicochemical characterization of the BLGF/ODEX hydrogel have previously been discussed, and it was reported that the materials exhibited acceptable structural and functional properties for applications in bone tissue engineering [11].

**FIGURE 1:**
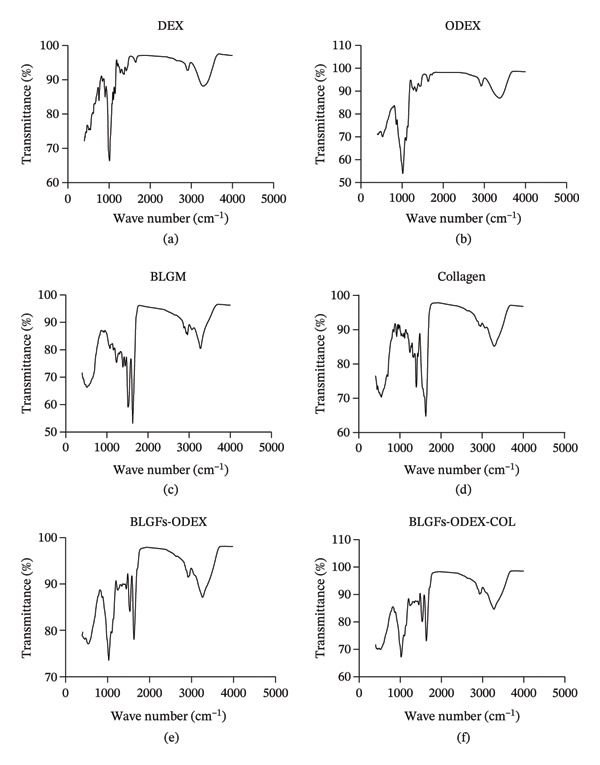
FTIR images of (a) dextran (DEX), (b) oxidized dextran (ODEX), (c) β‐lactoglobulin monomers (BLGM), (d) collagen, (e) β‐lactoglobulin fibres‐oxidized dextran (BLGFs‐ODEX), and (f) β‐lactoglobulin fibres‐oxidized dextran‐collagen (BLGFs‐ODEX‐COL).

From Figure 1a, b, there is no major difference in the FTIR spectra of DEX and ODEX, respectively. However, the appearance of a new aldehyde symmetric vibration band (carbonyl) at around 1730 cm^−1^ confirms the successful oxidation of dextran [15]. The analysis of BLGM FTIR spectra in Figure 1c reveals a characteristic peak around 1630 cm^−1^, which falls within the amide I region (1600–1700 cm^−1^). A peak at around 1690 cm^−1^ corresponds to turns, which are another secondary structure of proteins. These characteristic peaks are associated with β‐sheets and strands in β‐lactoglobulin. The peaks were retained in BLGFs‐ODEX at 1635 cm^−1^ (Figure 1e) and BLGFs‐ODEX‐COL at 1634 cm^−1^ (Figure 1f), indicating the preservation of the protein secondary structure. The FTIR image of ODEX (Figure 1b) shows a characteristic peak at around 1730 cm^−1^, which corresponds to the C=O aldehyde group. The peak disappeared in the BLGFs‐ODEX FTIR spectra (Figure 1e), which is an indication that it was consumed during cross‐linking. Nonetheless, the BLGFs‐ODEX FTIR spectra showed a new peak at around 1635 cm^−1^, which corresponds to a C=N stretching vibration, indicating the successful formation of a Schiff base bond between the amino and aldehyde groups. Collagen has been reported to have characteristic peaks in several absorption bands on the FTIR spectra. In Figure 1d, distinct characteristic peaks for collagen were visible at around 1560 cm^−1^ and 1243 cm^−1^, which represent amide II and III functional groups, respectively [16]. The characteristic peak of collagen at 1560 cm^−1^ disappeared in BLGFs‐ODEX‐COL (Figure 1f) while the peak at 1243 cm^−1^ was still visible. This is an indication of the successful incorporation of collagen in the biomaterial.

**FIGURE 2:**
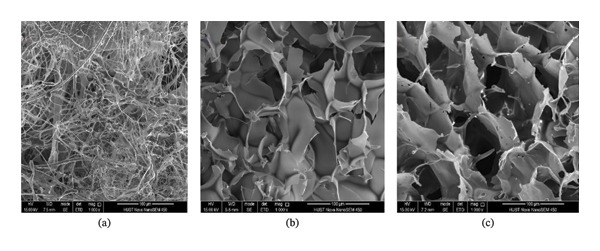
SEM images of collagen (a), β‐lactoglobulin fibres‐oxidized dextran (b), and β‐lactoglobulin fibres‐oxidized dextran‐collagen (c) which were freeze‐dried for 72 h.

The structural properties of COL, BLGFs‐ODEX, and BLGFs‐ODEX‐COL were analysed using a SEM (Nova NanoSEM 450), and the results are shown in Figure 2. It can be observed that SEM images of collagen (Figure 2a) showed a more fibrous structure than in BLFGs/ODEX and BLGFs/ODEX/COL (Figure 2b, c, respectively). BLFGs/ODEX and BLGFs/ODEX/COL SEM images revealed microporous structures with pore diameters averaging more than 20 μm. However, BLGFs/ODEX/COL showed a higher average pore diameter size than COL and BLFGs/ODEX. High porosity helps the biomaterials to absorb water and aids in oxygen circulation, which forms an important property of biomaterials for tissue engineering applications [17]. Having large pore sizes also allows stem cells to effectively migrate and proliferate within the biomaterial. The movement of nutrients and growth factors is much easier in a porous biomaterial. The results demonstrate that BLGFs/ODEX/COL can effectively support stem cell proliferation and differentiation by utilizing its porous structure to allow the cells, oxygen, nutrients, and growth factors to easily move through the biomaterial. This was equally reported in another study where biomaterial scaffolds with a high porosity were said to be better suited to cell migration, vascularization, and nutrient and waste transportation [18].

### 3.2. Cell Viability Assay

**FIGURE 3:**
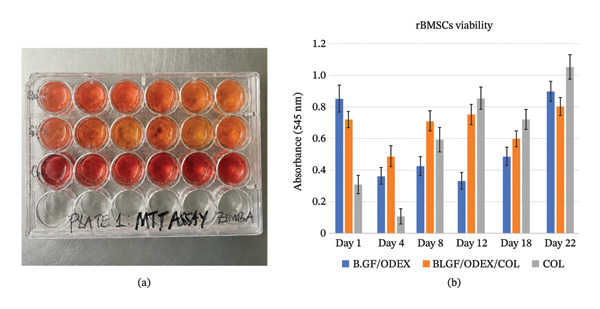
(a) rBMSCs cultured on BLGF/ODEX, BLGF/ODEX/COL, and COL using growth media. (b) The viability of rBMSCs on BLGF/ODEX, BLGF/ODEX/COL, and COL at various time intervals (the data presented are expressed as mean, *n* = 3).

To be considered suitable for tissue engineering applications, biomaterials must exhibit excellent cytocompatibility, particularly in supporting stem cell viability [19, 20]. rBMSCs’ viability on the synthesized test materials (BLGF/ODEX, BLGF/ODEX/COL, and COL) was analysed using the MTT assay. These as‐synthesized hydrogel materials were put in a 24‐well plate, as shown in Figure 3a, and rBMSCs were seeded on the hydrogels at a density of 2 × 10^3^ cells/well. The bar graphs presented in Figure 3b illustrate the viability of rBMSCs cultured on the hydrogel materials over a 22‐day period. The data demonstrate that all three hydrogel formulations (BLGF/ODEX, BLGF/ODEX/COL, and COL) were able to support cell viability throughout the entire culture period, indicating that the materials are noncytotoxic. Notably, on Day 1, cell viability on the BLGF/ODEX hydrogel was higher than on the COL hydrogel, suggesting a more favourable initial environment for cell attachment and survival. No marked difference in cell viability was observed between the BLGF/ODEX and BLGF/ODEX/COL groups on Day 1.

A general trend of decreased cell viability was observed across all materials on Day 4, followed by a marked recovery on Days 8 and 12 and a subsequent decline again on Day 18. Interestingly, the highest cell viability across all test groups was recorded on Day 22, suggesting long‐term biocompatibility and the potential of the materials to support sustained cell proliferation. These fluctuations in cell viability may be attributed to the dynamic nature of stem cell proliferation and the progressive remodelling of the hydrogel matrix, including changes in weight, porosity, and surface characteristics induced by ongoing cell‐material interactions and culture conditions [21].

Among the three tested hydrogel materials, BLGF/ODEX/COL consistently supported higher cell viability throughout the culture period. This improved performance of BLGF/ODEX/COL over BLGF/ODEX and COL can be attributed to the bioactivity of collagen extracted from *Chambo* tilapia fish skin, which provides binding sites for integrins, promotes ECM signalling, and provides a biomimetic microenvironment for rBMSCs [22]. This indicates that the incorporation of collagen enhanced the biological performance of the hydrogel. Collagen extracted from tilapia fish skin has been reported to exhibit excellent ability to support stem cell viability [23]. It can therefore be concluded that the remarkable difference in cell viabilities between BLGFs/ODEX and BLGF/ODEX/COL was mainly due to the presence of the collagen, which was extracted from *Chambo* tilapia fish skin. This suggests that BLGF/ODEX/COL holds the most promise for use in bone tissue engineering and other cell culture applications.

### 3.3. Cell Morphology Analysis on the Hydrogels

**FIGURE 4:**
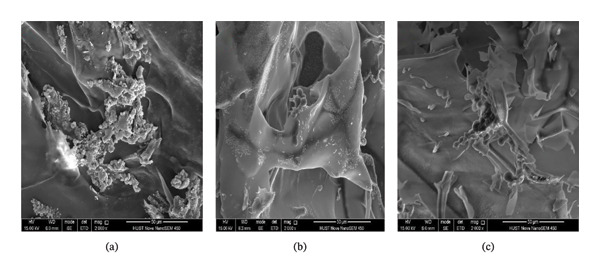
SEM images showing rat bone marrow stem cells cultured on collagen (a), β‐lactoglobulin fibres‐oxidized dextran (b), and β‐lactoglobulin fibres‐oxidized dextran‐collagen (c).

An SEM was used to observe the attachment and morphological structure of the rBMSCs cultured on hydrogel materials. SEM images in Figure 4 show that rBMSCs were able to adhere to all three hydrogel materials, which demonstrates that the hydrogels were able to support cell adhesion and proliferation. This is also an indication that the materials are non‐cytotoxic, as the cells were able to attach and proliferate on the surface of the biomaterials.

Although collagen is a biomaterial that supports cell attachment, it can be seen in Figure 4a that the rBMSCs were clustered with predominantly rounded morphology and limited spreading on the material. This suggests that there was poor substrate adherence and suboptimal cytoskeletal development. This could be attributed to the poor mechanical stability and rapid degradation of collagen, which can restrict long‐term cell proliferation and differentiation [24]. In contrast, better cell spreading was observed in BLGFs‐ODEX (Figure 4b), with rBMSCs showing visible filopodia and better interaction with the substrate. Enhanced cell adhesion may be attributed to the presence of BLGFs, which mimics the ECM since it is derived from animal protein [25]. The presence of ODEX further enhances the mechanical stability and hydrophilicity of the material, thereby improving its biocompatibility [26]. BLGFs‐ODEX‐COL (Figure 4c) demonstrated the most favourable conditions for osteogenic differentiation. The rBMSCs exhibited more extensive cytoskeletal spreading, elongated morphology, and robust filopodia extension bridging across the biomaterial, which is an indication of active matrix interaction. The *Chambo*‐derived collagen likely contributed to this by providing integrin‐binding motifs (Arg‐Gly‐Asp), facilitating enhanced cell‐matrix interaction [27]. It is evident that this hybrid biomaterial benefits from the functional properties of collagen and the structural integrity of BLGFs‐ODEX. This combination supports both cell attachment and early osteogenic differentiation signals, as it has been reported in similar biomaterials in other studies [28, 29]. These morphological observations from these SEM images suggest that the BLGFs‐ODEX‐COL biomaterial provides a better environment for bone tissue regeneration.

### 3.4. Haemocompatibility

One of the major requirements for the use of materials in vitro and in vivo experiments involving cells is the haemocompatibility of the materials. As such, animal blood cells ought to be subjected to extracts from the materials to determine if they will result in the lysis of the cells or not. Table 1 summarizes the average absorbance values that were obtained after incubating rat RBCs with extracts from the test samples. There was no major difference in the OD values between the test samples (BLGF/ODEX, BLGF/ODEX/COL, and COL), which indicates that all the materials exhibited good cytocompatibility.

**TABLE 1:** The absorbance values of the supernatants of the samples at OD_545_ nm.

BLGF/ODEX/COL	COL	BLGF/ODEX	PBS (−ve ctrl)	Deionised water (+ve ctrl)
0.114	0.0701	0.101	0.0733	0.543

**FIGURE 5:**
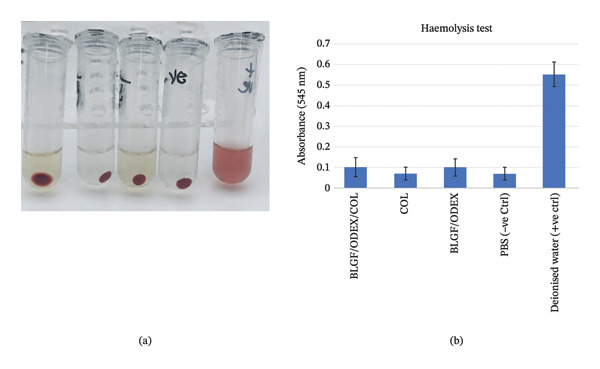
The results of the haemolysis test of rat red blood cells in the presence of the extracts from BLGF/ODEX, BLGF/ODEX/COL, COL, PBS, and deionised water. (a) Optical image of rat red blood cells incubated in sample extracts after centrifugation. (b) The haemolysis bar graph of the samples is plotted against the absorbance values at OD_545_ nm (the data presented are expressed as mean, *n* = 3).

A visual photograph following centrifugation of a mixture of rat RBCs and extracts from the test materials (BLGF/ODEX, BLGF/ODEX/COL, and COL) is shown in Figure 5a. Phosphate buffer solution (PBS) was used as a negative control, while deionised water was used as a positive control.

The results in Figure 5a showed that there was no indication of haemolysis amongst the test materials and the negative control, while there was clear evidence of RBC lysis in the positive control group (Deionised Water). There was minimal colour change in BLGF/ODEX, BLGF/ODEX/COL, COL, and PBS samples, which suggests low levels of haemoglobin release; while in the control group, there was intense red colouration indicating rapid erythrocyte rupture, which results in the release of haemoglobin. The hypotonic environment in the deionised water caused osmotic shock, which was responsible for the rupturing of the RBCs [30].

As shown in Figure 5b, the haemolysis rate from all biomaterials was less than 2%, which is lower than the 5% threshold value established by ASTM for blood‐contacting biomaterials [31]. Collagen and BLGF/ODEX had haemolysis rates lower than 1%, while BLGF/ODEX/COL had a haemolysis rate marginally above 1% but still way below 2%. The slight differences in the haemolysis rates of the biomaterial extracts could be explained by the subtle variations in surface roughness or their chemical composition, which might slightly influence their interaction with RBCs [32]. However, the low haemolysis rates in all test samples highlight their excellent haemocompatibility, which is a particularly important property for biomaterials for in vivo applications (i.e., bone tissue regeneration) where contact with blood is inevitable.

### 3.5. Osteogenic Differentiation of rBMSCs Seeded on the Hydrogels

**FIGURE 6:**
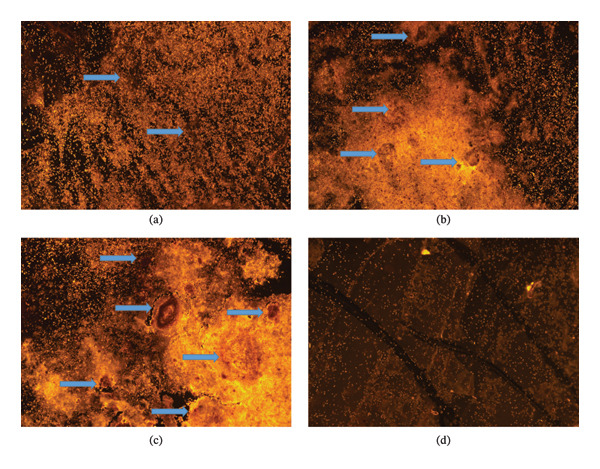
Alizarin red staining results showing osteogenic differentiation abilities of rBMSCs cultured on COL (a), BLGFs‐ODEX (b), BLGFs‐ODEX‐COL (c), and control group (d).

The osteogenic differentiation potential of rBMSCs on the hydrogel materials was evaluated using Alizarin Red (1%) staining, a well‐established detection method for calcium mineralization. Stem cell culture using growth media in the absence of biomaterial scaffolds was used as a control. After culturing the stem cells on the hydrogel for 14 days using osteogenic differentiation medium, the rBMSCs demonstrated clear evidence of osteogenesis. During Alizarin Red staining, the dye selectively binds to calcium deposits, and the presence of distinct orange‐red staining in the samples confirmed successful ECM mineralization. There was a considerable orange‐red staining in BLGFs‐ODEX (Figure 6b) than in COL (Figure 6a) biomaterials. Calcium deposits were less pronounced in COL samples, suggesting that while collagen can promote cell adhesion, it lacks essential structural and biochemical cues to independently support robust osteogenic differentiation of stem cells. On the other hand, the BLGFs‐ODEX‐COL (Figure 6c) biomaterial revealed the most intense and widespread Alizarin Red staining as evidenced by the dense orange‐red colouration, which suggests a higher degree of ECM mineralization, reflecting a better osteogenic differentiation. The collagen derived from *Chambo* tilapia fish acts as a bioinductive component that provides osteogenic signals and supports the deposition of calcium. Its resemblance to natural ECM proteins and its role in modulating osteoblast differentiation are well documented [33]. The inclusion of collagen in BLGFs‐ODEX‐COL likely creates a synergistic effect in the provision of a conducive microenvironment for osteogenic differentiation [34].

As shown in Figure 6a–c, the arrows highlight localized calcium deposits, further verifying the rBMSCs′ ability to undergo osteogenic differentiation and form bone‐like tissue. These findings suggest that the synthesized hydrogel materials effectively support osteogenesis, reinforcing their potential in bone regeneration applications. The results of the Alizarin Red staining in Figure 6 revealed that all the hydrogels were able to support the differentiation of rBMSCs into osteogenic tissue resembling bone tissue. Calcified tissue was visible in all the test materials (COL, BLGF/ODEX, and BLGF/ODEX/COL), which is an indication that the materials were able to support osteogenesis. Studies have used similar osteogenic differentiation media, and rBMSCs successfully differentiated into calcified tissue with the help of osteogenic growth factors present in the media [35, 36]. Calcification was more pronounced in the BLGF/ODEX/COL hydrogel, followed by the BLGF/ODEX hydrogel, and it was least in the COL hydrogel, suggesting that the BLGF/ODEX/COL hydrogel is better suited for applications in bone tissue engineering. Surely the presence of collagen in BLGF/ODEX/COL hydrogel was the difference as compared to BLGF/ODEX hydrogel. Collagen is one of the most common components of body organs, including bone tissue. Collagen has also been reported as a biomaterial that enhances bone tissue regeneration [37].

It is evident that collagen was successfully extracted from *Chambo* tilapia fish skin using acetic acid and salt precipitation. The choice of marine‐sourced collagen, particularly from tilapia, was strategic, as it offers notable advantages over mammalian collagen. Marine‐sourced collagen presents a reduced risk of zoonotic disease transmission, cultural acceptance, and sustainability. Besides, tilapia‐derived collagen is predominantly type I, which is also the primary collagen that is found in mammalian bone, skin, and tendons [38]. This similarity enhances its biological relevance and effectiveness in tissue engineering applications. Moreover, collagen extracted from tilapia fish exhibits excellent biocompatibility, biodegradability, and low immunogenicity [9]. These characteristics allow it to integrate well with host tissues without eliciting a significant immune response and support key cellular functions like adhesion, proliferation, and osteogenic differentiation. In the context of this study, the inclusion of *Chambo*‐derived collagen in the hydrogel enhanced the structural and functional performance of the resulting biomaterial. Its presence contributed to improved cell interaction on the material, calcium deposition, and overall regenerative potential of the BLGFs/ODEX/COL hydrogel, making it an ideal candidate for bone tissue engineering applications.

The findings in this study complement our previous study, which established the favourable properties of the BLGFs/ODEX/COL hydrogel [11]. The present work further demonstrates that these properties translate into enhanced cell adhesion, haemocompatibility, and osteogenic differentiation capabilities as compared to other hydrogels. The performance of BLGFs/ODEX/COL hydrogel was better as compared to BLGFs/ODEX and COL hydrogels individually. By comparison, Jha et al. [39] had to enhance the osteogenic differentiation capability of human mesenchymal stem cells (hMSCs) that were cultured on a poly(N‐isopropylacrylamide) (p(NIPAAm)) hydrogel system by increasing the adhesive peptide density of the substrate, while BLGFs/ODEX/COL hydrogel was able to achieve remarkable osteogenic differentiation on its own. Furthermore, the addition of COL from tilapia fish offers a sustainable and cost‐effective alternative to mammalian collagen, plus they all have excellent biocompatibility [40]. Other bone graft substitutes like HA and calcium phosphate cements do offer excellent mechanical support, but they lack biological cues that are essential in tissue engineering [41, 42].

## 4. Conclusion

This study developed a functional biomaterial that not only offers structural support but also enhances biological activity for bone tissue regeneration. By combining BLGFs, ODEX, and collagen extracted from *Chambo* tilapia fish skin, we were able to synthesize hydrogel biomaterials with promising characteristics. Material characterization confirmed successful cross‐linking and a highly porous structure, with the BLGFs‐ODEX‐COL hydrogel showing better integrity and pore size, which is important for nutrient flow and cell migration. In vitro analysis confirmed that all the synthesized biomaterials were biocompatible and supported the proliferation of rBMSCs, with the BLGFs/ODEX/COL hydrogel showing enhanced performance due to the presence of tilapia‐derived collagen, which largely improved the biological performance of the hydrogel. SEM images further confirmed excellent cell attachment and spreading, especially on the BLGFs/ODEX/COL hydrogel, which supports early osteogenic signals. Haemocompatibility tests showed haemolysis rates under 2% for all the hydrogels, indicating their safety for in vivo use. Moreover, Alizarin Red staining demonstrated strong mineralization in rBMSCs cultured on the biomaterials, with the BLGFs‐ODEX‐COL hydrogel outperforming the others in promoting calcified matrix formation. Overall, these results underscore that the incorporation of collagen extracted from *Chambo* tilapia fish skin was a key factor in enhancing the structural and functional performance of the biomaterial. The BLGFs/ODEX/COL hydrogel biomaterial stands out as a safe and effective scaffold with great potential for bone tissue engineering, especially in resource‐limited settings where cost‐effective and accessible biomaterials are critically needed.

## Author Contributions

Bhahat Lawlley Zimba: conceptualization, methodology, formal analysis, and writing–original draft. Xiaohan Yang: data curation, formal analysis, and writing–review and editing. Mwemezi Rwiza and Elingarami Sauli: data curation, and writing–review and editing. Shenqi Wang: data curation, writing–review and editing, and supervision.

## Funding

The authors did not receive any funding in coming up with this research paper.

## Conflicts of Interest

The authors declare no conflicts of interest.

## Data Availability

Data for this study are available upon a formal request to the corresponding author.
